# Novel Behavior of the 2019 Novel Coronavirus With Invasion of the Cardiac Conduction System in the Young

**DOI:** 10.7759/cureus.11115

**Published:** 2020-10-23

**Authors:** Abdullah A Alabdulgader, Abdulrhman Alabdulgader, Metin Sungur, Hesham Essa, Khalid Al Khamees

**Affiliations:** 1 Congenital Heart Service, Prince Sultan Cardiac Center, Al-Hasa, SAU; 2 Cardiology, College of Medicine, King Faisal University, Al-Hasa, SAU; 3 Cardiology, Prince Sultan Cardiac Center, Al-Hasa, SAU; 4 Cardiovascular Surgery, Prince Sultan Cardiac Center, Al-Hasa, SAU

**Keywords:** complete heart block, covid-19 and heart, permanent pacemaker implantation (ppm)

## Abstract

We report the case of a 35-year-old man (an oil engineer) referred as a coronavirus disease-2019 (COVID-19) case with heart block and a four-day history of headache and fever. The patient was hemodynamically stable with normal respiratory effort and oxygen saturation. Three consecutive COVID-19 tests were positive since admission. Comprehensive clinical assessment investigations were performed. Apart from mild acute phase reactants elevation, all results were within reference limits. He had no leukocytosis and normal cardiac enzymes, chest x-ray findings, echocardiography findings, and healthy coronary arteries. The patient had a fever and electrocardiographic evidence of sinus node dysfunction associated with Mobitz type 2 atrioventricular block that progressed to complete heart block. This was a unique presentation of COVID-19 in a young, otherwise healthy man with the sole manifestation confined to the cardiac conduction system and the absence of respiratory involvement, hypoxemia, and acidosis.

## Introduction

On January 7, 2020, a novel coronavirus, originally abbreviated as 2019-nCoV by the World Health Organization (WHO), was identified from a throat swab sample [1]. This pathogen was later renamed the severe acute respiratory syndrome-coronavirus-2 (SARS-CoV-2) by the Coronavirus Study Group [2], and the disease was named coronavirus disease 2019 (COVID-19) by the WHO. Based on the report of the first 425 confirmed cases in Wuhan, common symptoms include fever, dry cough, myalgia, and fatigue; less common symptoms are sputum production, headache, hemoptysis, abdominal pain, and diarrhea [3]. A descriptive, exploratory analysis of the first 72,314 cases of COVID-19 revealed that cardiovascular involvement was reported in just 10.5% of cases, but it was never the sole manifestation [4]. We present the case of COVID-19 affecting the cardiac conduction system of a young man in the absence of typical symptoms, confounders, and comorbidities. This case offers a unique opportunity to investigate the cellular pathogenicity of COVID-19.

## Case presentation

The patient is a 35-year-old man and a petroleum engineer. He was doing very well with no significant previous medical history of note. He had a history of contact with a COVID-19-positive campmate. There was no history of travel to tropical countries, no family history of heart block or sudden death, neither a previous history of jaundice nor a history of skin rashes. He sought medical advice four days before his referral to our cardiac center because of headache and fever. He was admitted into the coronary care unit into a single negative pressure room and under strict droplet isolation precaution measures. Clinical evaluation revealed a temperature of 38°C, which subsided with one dose of antipyretic. Otherwise, he had stable vital signs and hemodynamics. His oxygen saturation was 98% on room air. His body weight was 85 kg, with a height of 170 cm (body mass index, 29.4 kg/m2). He was found to have severe bradycardia with a heart rate of 33 beats per minute (BPM), with otherwise healthy vital signs and cardiac examination results. He had symptoms of COVID-19-fever and headache. Before initiation of hydroxychloroquine the patient has baseline ECG, which was normal and screened for glucose 6-phosphate dehydrogenase deficiency and it was normal. He was treated with a course of hydroxychloroquine 400 mg orally for the first dose, then 200 mg twice daily for seven days. He also received levofloxacin 200 mg orally for five days and vitamin C 500 mg orally twice daily. On the third day after admission, he developed a skin rash and diarrhea, but both conditions subsided spontaneously. Serial laboratory tests, including a complete blood count and blood differential counts, serum electrolytes, liver function test, renal function test, lipid profile, and arterial blood gas, were within reference ranges. His coagulation profile and cardiac enzymes were both within reference ranges during the first day of admission. Liver functions increased at five days of admission due to possible hydroxychloroquine side effect. Acute phase reactants showed mildly increased C-reactive protein of 2.27 mg/dl (reference range of 0-0.8 mg/dl), erythrocyte sedimentation rate of 42 mm/h, and ferritin level of 525 ng/ml (reference range of 30-400 ng/ml), with healthy low-density lipoprotein (LDL) in the absence of leukocytosis. Arterial blood gas confirmed neither absence of acidosis nor any other acid-base imbalance disorders. Three serial tests of COVID-19 were positive in the first 21 days. Laboratory investigations were performed, as shown in Table 1.

**Table 1:** Serial laboratory tests. WBC, White blood cell; RBC, red blood cell; HB, hemoglobin; HCT, hematocrit; BUN, blood urea nitrogen; Na, sodium; K, potassium; AST, aspartate aminotransferase; ALT, alanine aminotransferase; T. BIL, total bilirubin; CBC, complete blood count; LDL, low-density lipoprotein; CRP, C-reactive protein; ESR, erythrocyte sedimentation rate; PT, prothrombin time; PTT, partial thromboplastin time; INR, international normalized ratio.

Date	CBC					Renal Function Test		Serum Electrolyte		Liver Function Test		
	WBC (10 x 3/μl)	RBC (10 x 6/μl)	HB (g/dl)	HCT (%)	PLATELET (10 x 3/μl)	BUN (mmol/L)	CREA (μmol/L)	NA (mmol/L)	K (mmol/L)	AST (U/L)	ALT (U/L)	T. BIL (μmol/L)
18/05/2020	6.18	5.52	15.4	44	191	4.6	95	134	3.88	18.6	19.8	28.9
19/05/2020	6.15	5.68	15.5	45.6	179	3.2	99	138	4.1	21	30	30.4
21/05/2020	6.44	5.57	15.4	43.8	132	2.8	96	139	4.2	37	47	54.1
23/05/2020	6.45	5.55	15.2	43.4	131	2.4	87	138	3.9	64	88	42.6
25/05/2020	6.10	5.26	14.3	40.6	169	1.8	83	137	3.4	72	136	29.5
27/05/2020	8.18	5.59	15.2	43.1	220	2.4	104	138	4.2	42	110	
31/05/2020	7.6	5	13.8	39.6	281	2.2	89					
	WBC Differential Count (per μl)	Count		COVID-19 Confirmatory Test								
21/05/2020	Neutrophil%	55.6		16/05/2020	Positive							
	Lymphocyte%	38.4		23/05/2020	Positive							
	Monocyte%	5.5		28/05/2020	Positive							
	Eosinophil%	0.3										
	Basophil%	0.2										
	Lipid			Additional Tests								
	Cholesterol (mmol/l)	LDL (mmol/L)	Triglyceride (mmol/L)	CRP (mg/dl)	ESR (mm/hr)	PT (sec)	PTT (sec)	INR (sec)	LDH (U/L)			
18/05/2020	2.91	1.36	1.55						178			
19/05/2020	2.8	1.2	1.59		12							
21/05/2020	2.8	1.3	1.41									
23/05/2020	2.6	1	1.69	2.27								
25/05/2020	2.5	1.1	1.18		13.5	35.7	31	5.25				

**Figure 1:**
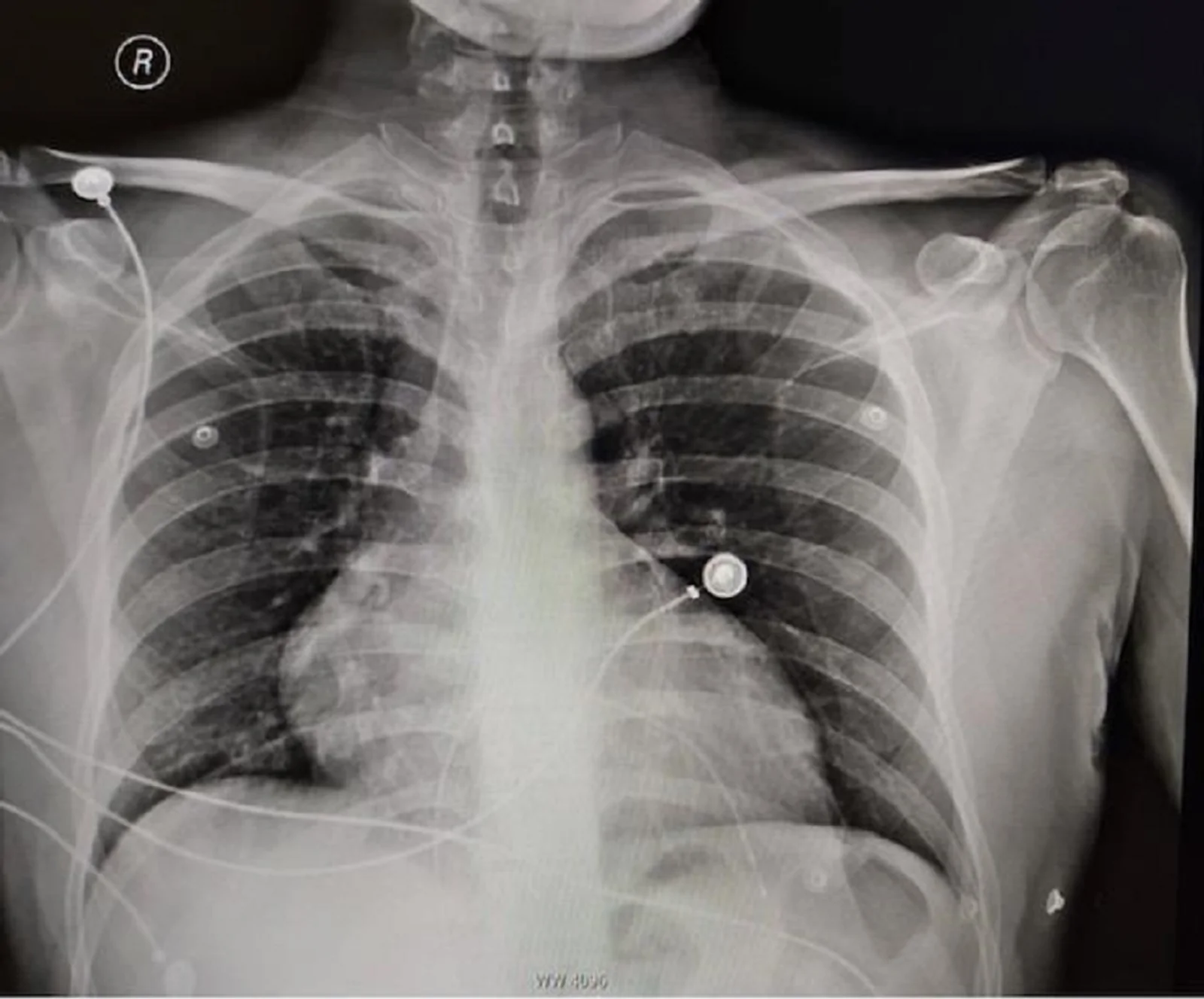
Chest radiograph obtained after insertion of a temporary transvenous pacing lead via the right femoral vein. The lung fields are clear; the temporary pacing lead is visible.

**Figure 2:**
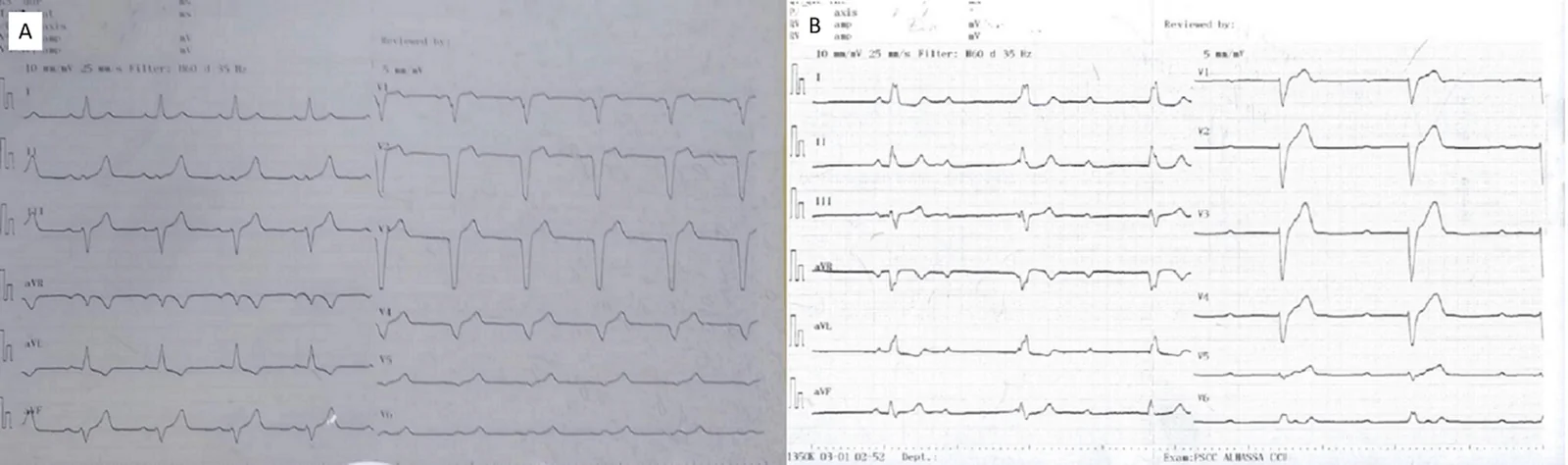
(A) A 12-lead electrocardiogram with sinus rhythm at the beginning of the disease. (B) A 12-lead electrocardiogram illustrating widespread insult to the cardiac conduction system: a complete heart block with a ventricular rate of 32-42 beats per minute with evidence of sinus node dysfunction and interventricular conduction block.

**Figure 3:**
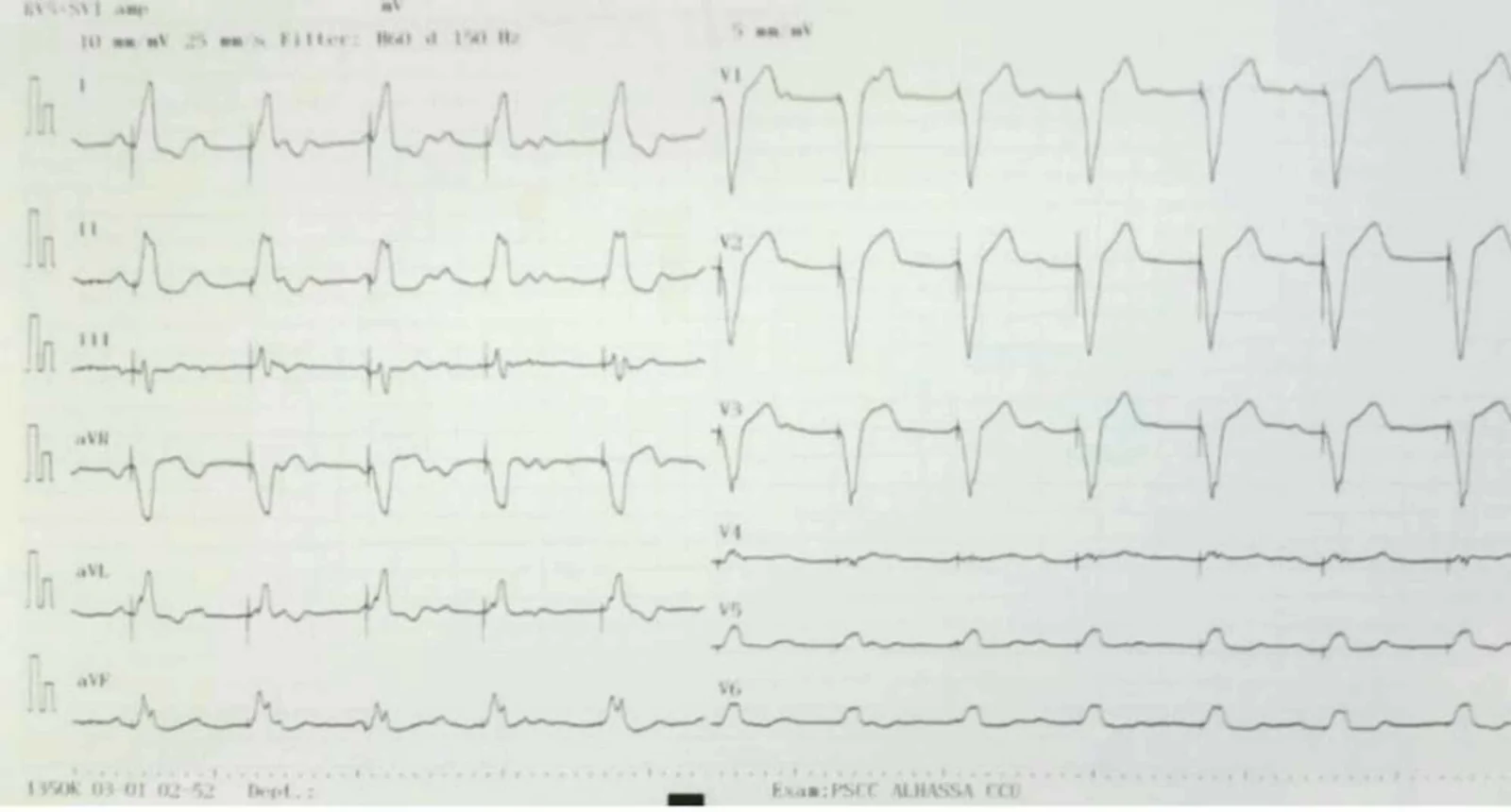
A temporary emergency pacemaker was implanted with a pacing rate of 75 beats per minute.

**Figure 4:**
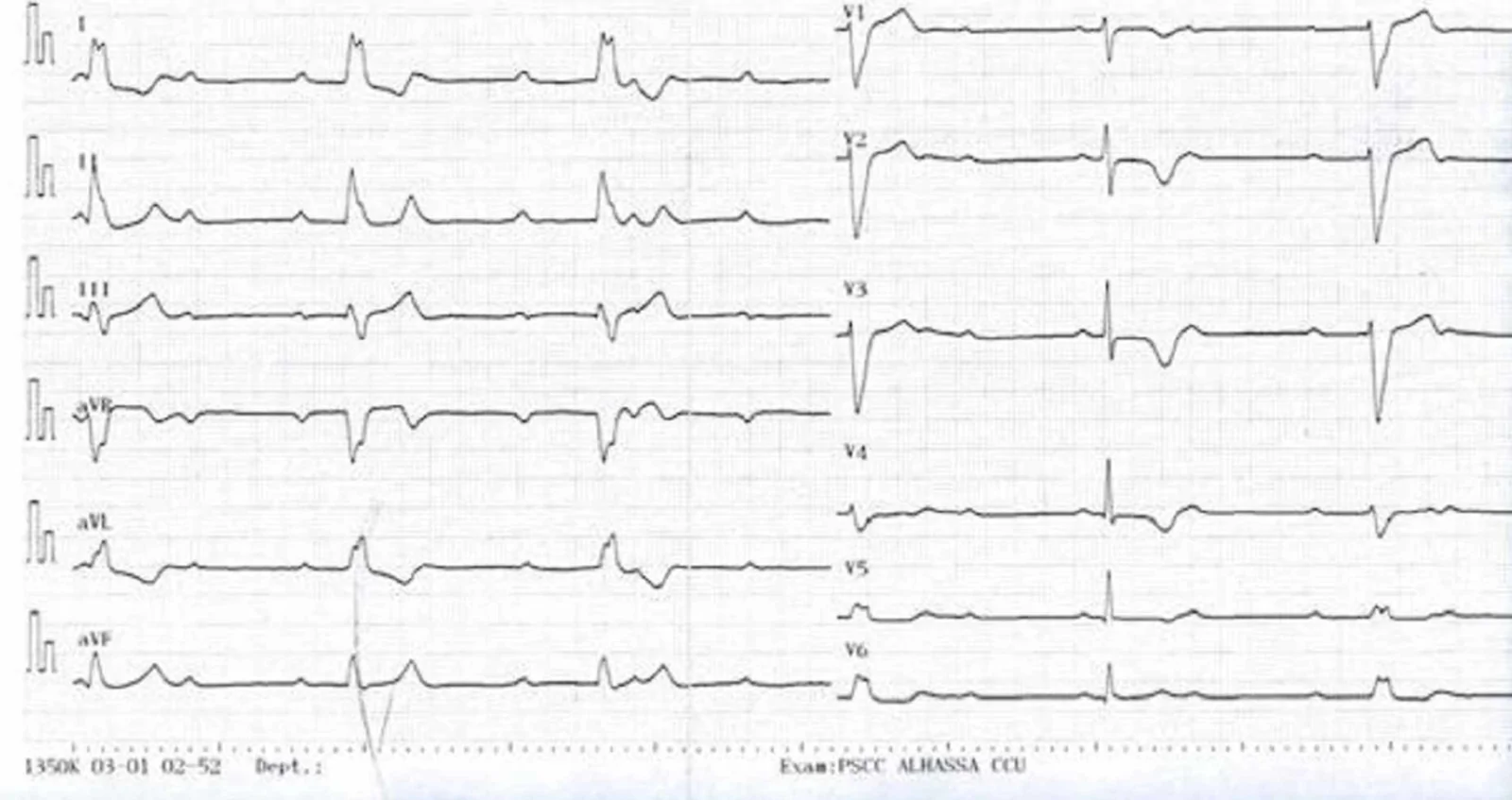
After five days of the cardiac conduction system insult, occasional normally conducted sinus beats can be seen, giving hope of possible recovery and may eliminate the need for a permanent pacing system.

**Figure 5:**
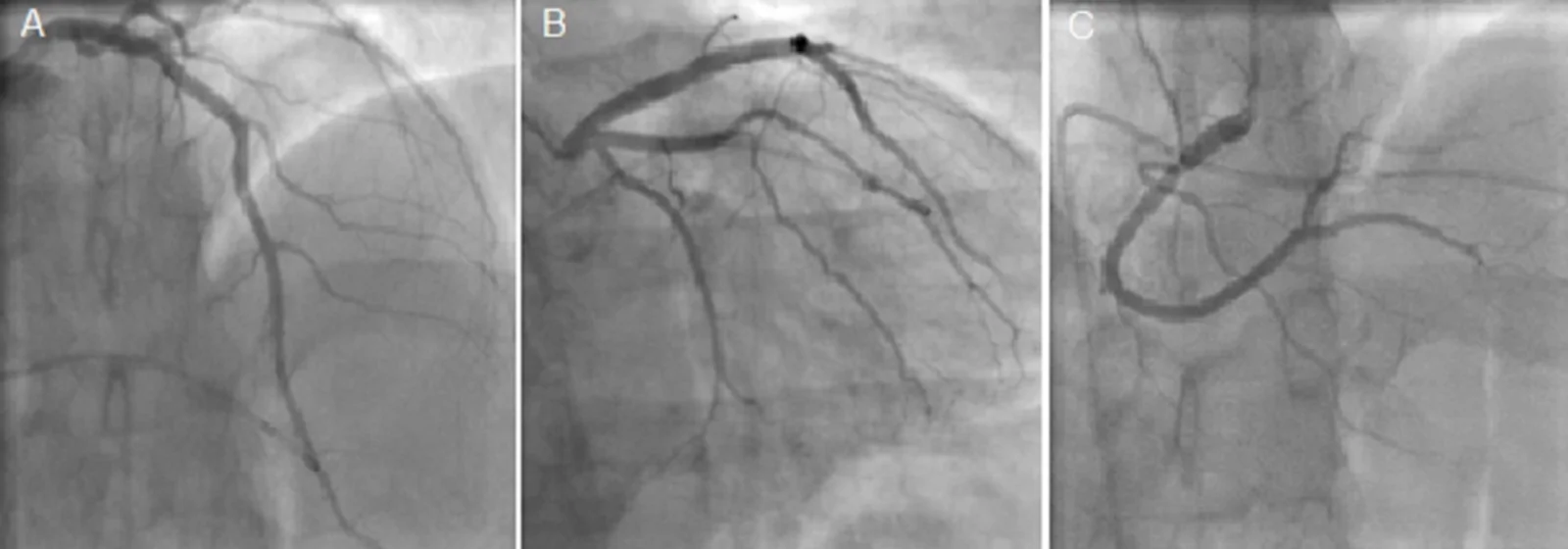
(A) An anteroposterior cranial view of the left coronary system shows a normal left main coronary artery, left anterior descending artery, and left circumflex artery. (B) The right anterior oblique caudal of the left coronary system shows a normal left main artery, left anterior descending artery, and left circumflex artery. (C) An anteroposterior cranial view of the right coronary system shows a normal right coronary artery.

**Figure 6:**
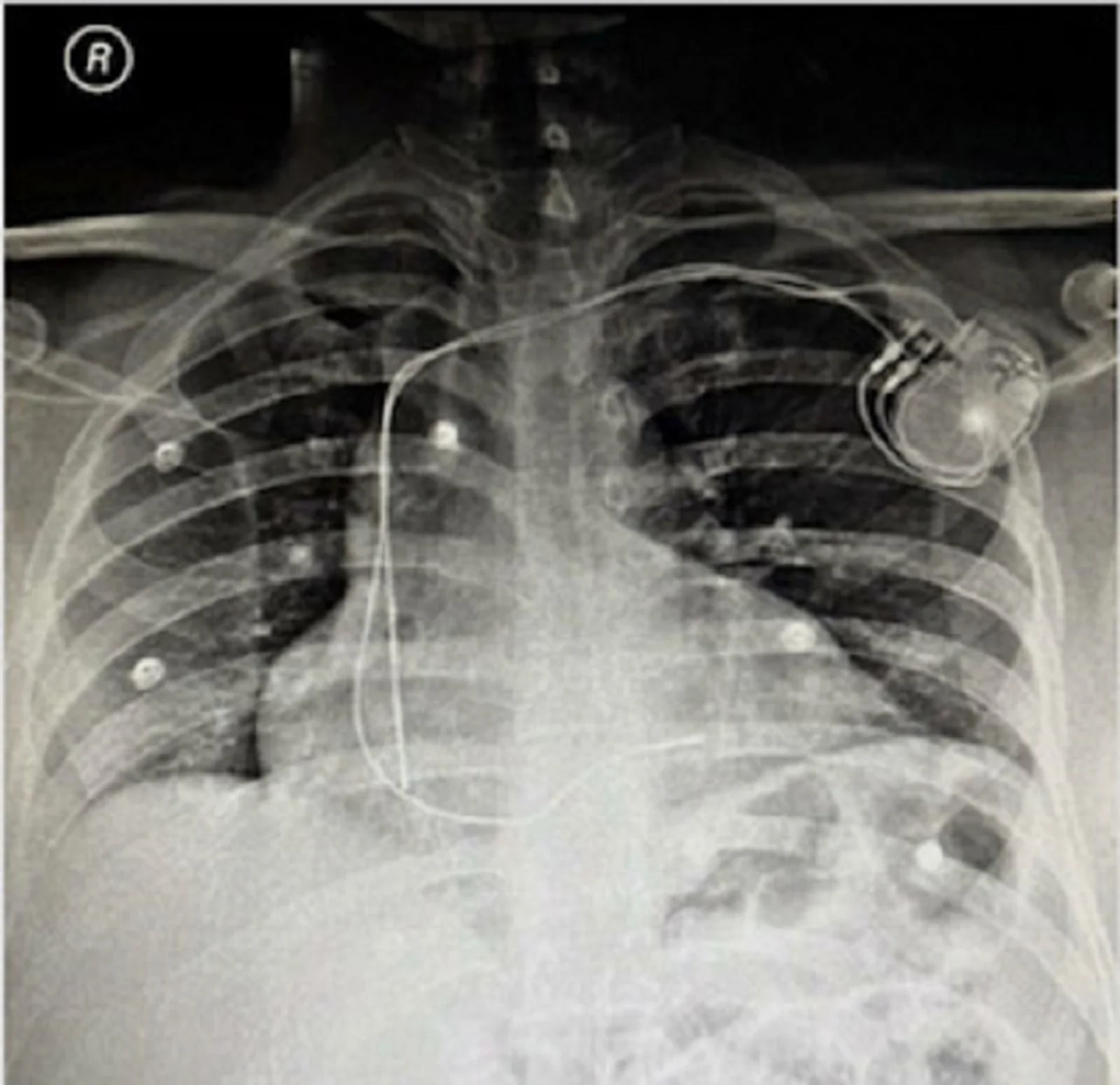
After 18 days, a dual-chamber pacemaker was implanted.

A chest x-ray obtained after temporary transvenous pacemaker insertion showed normal bronchovascular markings with free costophrenic angles (Figure 1). Serial electrocardiograms with Holter monitoring and telemetry records revealed second-degree atrioventricular conduction block (AVB; Mobitz type 2), which progressed to complete AVB. His ventricular rate fluctuated between 32 and 42 BPM with evidence of sinus node dysfunction and left bundle branch block (Figures 2A, 2B). A temporary pacemaker was implanted immediately to support circulation (Figure 3). There were trials to examine the intrinsic heart rate and evaluate his conduction system status. Evaluation of intrinsic rate after five days of admission revealed scattered normally conducted sinus beats, which was interpreted as a possible recovery of the cardiac conduction system (Figure 4). Cardiac catheterization revealed healthy coronary arteries (Figure 5). As there is no clear-cut reference period in the current medical literature for the maximum time before implanting a permanent pacing system in a COVID-19-based heart block, we planned for a three-week waiting time. This is one week more than our reference waiting time after an atrioventricular (AV) block post cardiac surgery. After 18 days, the temporary system site was inflamed, causing pain and distress. The team decided to implant a permanent dual-chamber system on the same day (Figure 6). During the third month after pacemaker implantation, the patient was still having complete AV block and was considered a stage four dependency (pacemaker-dependent).

## Discussion

SARS-CoV-2 is a member of the family Coronaviridae and order Nidovirales. The family consists of two subfamilies: Coronavirinae and Torovirinae. The first 425 confirmed cases in Wuhan established common symptoms that consisted of fever, dry cough, myalgia, and fatigue, and less common symptoms were sputum production, headache, hemoptysis, abdominal pain, and diarrhea [3]. An analysis of the first 72,314 cases of COVID-19 revealed cardiovascular involvement in 10.5% of cases, and never as the sole manifestation [4]. In severe cases, the electrophysiological disturbances of the heart occur in up to 44% of cases [5]. Myocardial injury, as defined by an increased troponin level, can occur in COVID-19-affected individuals due to myocardial ischemia or non-ischemic myocardial processes, including myocarditis [6,7]. The high prevalence of arrhythmic events in affected patients might be contributed to diverse metabolic derangements like hypoxia, or neurohormonal or inflammatory stress in the presence of viral infection in patients with or without prior cardiovascular disease [8]. One study reported that 26% of patients hospitalized at Zhongnan University Hospital of Wuhan required cardiovascular intensive care. Of these, 16.7% developed arrhythmias, and 7.2% had acute coronary syndrome. The involvement of the cardiac conduction system is almost always associated with respiratory involvement, with an average age of 55 years, and occurs predominantly in men. More than 73% of deaths occur in patients older than 65, with a striking paucity of pediatric deaths (0.06%) [5]. COVID‐19 is a systemic infection that prominently impacts the hematopoietic system and hemostasis. Lymphopenia is an important measure of the blood differential count with prognostic potential. Neutrophil/lymphocyte ratio and peak platelet/lymphocyte ratio are also important prognostic indicators in severe cases [9]. Serial longitudinal measurements of differential blood counts are important to treatment planning and follow-up. Terpos et al. described three cases of COVID-19 and cardiac arrhythmias [10]. All three cases were in patients of advanced age with specific rhythm disorder compared to our case, a 35-year-old man with evidence of widespread conduction system insult. This suggests a higher level of affinity between the SARS-CoV-2 and cellular components of the cardiac conduction system. Also the pathomechanism seems to be different because all three cases suffered from respiratory involvement and hypoxemia as well as other comorbidities like diabetes mellitus and systemic hypertension [10]. Our patient is an otherwise healthy young man. 

Peigh et al. reported two cases with sinus node dysfunction; both in older patients (one aged 70 years, and one aged 81 years) with severe hypoxemia, who required intubation and mechanical ventilation. Both patients had ascending aortic aneurysm, hypertension, and obstructive sleep apnea [11]. A peculiar conduction system insult was documented long ago in rats due to hypoxia and/or acidosis [12]. The absence of hypoxia and/or acidosis in our case may indicate other hidden risk factors and pathomechanisms of interaction at the cellular level. 

This case presentation is unique in the medical literature. Our 35-year-old man presented with evidence of widespread pathology of the cardiac conduction system with evidence of sinus node dysfunction, atrioventricular node dysfunction, and interventricular conduction block. This pathology was found to be highly specific to the cardiac conduction system, sparing other parts of the heart like the coronary arteries or myocardium along with other body systems, notably, the respiratory system. Myocarditis is a special concern with this scenario of presentation but was found to be remote and unlikely diagnosis due to absence of inflammatory markers increase, normal cardiac enzymes as well as preserved cardiac functions. In addition, there was no pericardial effusion. The available literature in similar viral infections assumes that cardiac pathology in humans is directly related to respiratory tract damage and a subsequent inflammatory reaction that may lead to cardiac complications in patients with significant comorbidities [13,14]. To the best of our knowledge, no similar case has been described in the literature since COVID-19 was identified. This very remarkable conduction tissue specificity occurs with evidence of continuous viremia and virus shedding, given the persistence of positivity of three tests for COVID-19 over the patient’s full admission period. 

The hematological picture revealed only a mild inflammatory response. In the absence of clear guidelines of the maximum time to allow for the AVB to recover, we deferred the decision of a permanent pacemaker and monitored the conduction system for possible recovery. We speculate that this novel virus traumatizes the specialized conduction cardiomyocytes, including the Purkinje cells, in a manner similar to the influenza-A virus, even without apparent respiratory damage [15]. Filgueiras-Rama et al. documented severe bradycardia and conduction abnormalities in one recombinant virus carrying PA D529N (PAmut)-infected mouse with the influenza-A virus. Viral pathogenicity determines early inflammation and imbalance in the extracellular matrix of infected rat hearts. Infected animals show active viral replication in cardiac Purkinje cells [16]. Further studies are needed to assess virulence factors and the host genes of SARS-CoV-2 that allow the virus to interact and damage the cardiac conduction system in humans. 

## Conclusions

A 35-year-old man presented with evidence of widespread pathology of the cardiac conduction system. This pathology was found to be highly specific to the cardiac conduction system, sparing other parts of the heart like the coronary arteries or myocardium along with other body systems, notably, the respiratory system. Severe damage to the conduction system in permanent conduction abnormalities can be rare as the primary manifestation of COVID-19 infection. The highly specific affinity of SARS-CoV-2 and its infective capacity to the cardiac conduction system in our case may act as a strong correlation for future histopathological, immunogenic, and cellular and molecular alteration investigations toward achieving successful therapeutic and preventive measures against COVID-19 in humans.
