# Correction: Novel Behavior of the 2019 Novel Coronavirus With Invasion of the Cardiac Conduction System in the Young

**DOI:** 10.7759/cureus.c484

**Published:** 2026-10-02

**Authors:** Abdullah A Alabdulgader, Abdulrhman Alabdulgader, Metin Sungur, Hesham Essa, Khalid Al Khamees

**Affiliations:** 1 Congenital Heart Service, Prince Sultan Cardiac Center, Al-Hasa, SAU; 2 Cardiology, College of Medicine, King Faisal University, Al-Hasa, SAU; 3 Cardiology, Prince Sultan Cardiac Center, Al-Hasa, SAU; 4 Cardiovascular Surgery, Prince Sultan Cardiac Center, Al-Hasa, SAU

This article has been corrected to revise the Figure 1 legend and its introducing sentence to clarify that the chest radiograph was obtained after insertion of a temporary transvenous pacing lead, which is visible in the image, and to remove the description of the cardiac silhouette as normal.

